# Randomised trial investigating the relationship of response rate for blood sample donation to site of biospecimen collection, fasting status and reminder letter: The 45 and Up Study

**DOI:** 10.1186/1471-2288-12-147

**Published:** 2012-09-24

**Authors:** Emily Banks, Nicol Herbert, Kris Rogers, Tanya Mather, Louisa Jorm

**Affiliations:** 1 National Centre for Epidemiology and Population Health, Australian National University, Canberra, Australia; 2 The Sax Institute, Sydney, Australia; 3 School of Medicine, University of Western Sydney, Sydney, Australia

**Keywords:** Biobank, Response rate, Fasting status, Reminder, Biospecimens

## Abstract

**Background:**

Various options exist for collecting biospecimens and biomarkers from cohort study participants, and these have important logistic, resource and scientific implications. Evidence on how different collection methods affect participation and data quality is lacking. This parallel-design randomised trial, the Link-Up Study, involved blood sample donation and other data collection among participants in an existing cohort study, The 45 and Up Study. It aimed to investigate the relation of fasting status, reminder letters and data collection site to response rates, data quality and biospecimen yield.

**Methods:**

Individuals aged 45 and over participating in The 45 and Up Study and living ≤20 km from central Wagga Wagga, NSW (regional area) or ≤10 km from central Parramatta, NSW (urban area) (n = 2340) were randomised, stratified by area of residence, to be invited to give a blood sample and additional data by attending either a clinic established specifically for the trial, with an appointment time (“dedicated clinic”, n = 1336) or an existing local commercial pathology centre (n = 1004). Within dedicated clinic groups, participants were randomised into fasting (n = 668) or non-fasting (n = 668) and, at the Parramatta pathology centre site, reminder letter after two weeks (n = 336) or no reminder (n = 334).

**Results:**

Overall, 33% (762/2340) of invitees took part in the Link-Up Study; 41% (410/1002) among regional and 26% (352/1338) among urban-area residents (p < 0.0001). At the dedicated clinics, response rates were 38% (257/668) not fasting and 38% fasting (257/668) (participation rate ratio (RR) = 1.00, 95%CI 0.91-1.08, p = 0.98). The response rate was 22% among individuals randomised to attend the Parramatta pathology centre without a reminder and 23% among those sent a reminder letter (RR = 1.01, 0.93-1.09, p = 0.74). In total, the response rate was 38% (514/1336) at the dedicated clinics and 25% (248/1004) at the pathology centres (RR = 0.67, 0.56-0.78, p < 0.01); measures of height, weight and systolic and diastolic blood pressure did not vary materially between these groups, nor did the median number of aliquots of plasma, buffy coat and red cells collected.

**Conclusions:**

Among cohort study participants, response rates for an additional study involving biospecimen collection, but not data quality or average biospecimen yield, were considerably higher at dedicated clinics than at existing commercial pathology sites.

## Background

Large-scale prospective studies have contributed enormously to our understanding of health. Increasingly, such large scale studies include the collection of biospecimens, to allow investigation and integration of genetic and other biomarker data within epidemiological research. There are multiple possible methods for the collection of biospecimens, ranging from in-home collection, use of mobile vans, attendance at a facility set up specifically for the purpose (termed here a “dedicated clinic”) [1], to use of existing health care facilities [2]. The choice of collection method has important logistic, resource and scientific implications. However, empirical evidence regarding the impact of different biospecimen collection methods on measures such as response rate and data quality is lacking.

This paper describes a randomised trial in Australia, comparing biospecimen and biomarker data collection (including physical measures) through dedicated clinics, set up specifically for the trial, with collection through existing commercial pathology centres. Specifically, the trial aimed to compare the response rate to an invitation to give a blood sample and have physical measurements (height, weight, blood pressure and heart rate) taken and the data quality and biospecimen yield, among existing participants from a population-based cohort study (The 45 and Up Study), in individuals randomised to:

1.Whether the participant was asked to attend fasting or not fasting;

2.Whether or not a reminder letter was sent to non-attending participants and;

3.Different sites of data collection (dedicated clinic *versus* an existing commercial pathology centre);

## Methods

### Study population

The “Link-Up Study” was conducted among a subset of 2340 individuals already taking part in The 45 and Up Study, a large scale study of healthy ageing of men and women aged 45 years and over from the general population of New South Wales, Australia. A total of 266,848 participants joined The 45 and Up Study between February 2006 and November 2008 by completing a postal questionnaire and providing written consent for long term follow-up of their health, including linkage to routinely collected population databases of health information. The 45 and Up Study is described in detail elsewhere [3].

### Data collection centres

An urban and a regional location were selected for this randomised trial, to allow comparison of response rates in these two types of location. The locations of Parramatta, New South Wales (urban) and Wagga Wagga, New South Wales (regional) were chosen on the basis of having over 1000 45 and Up Study participants resident in the target area, being fairly typical of urban and regional areas in New South Wales and having a local pathology centre. At each location, two collection sites were established; a dedicated clinic was set up *de novo* and a suitable commercial pathology centre was identified, with 4 collection sites established in total.

### Intervention

Eligible individuals were 45 and Up Study cohort members, resident in Wagga Wagga or Parramatta. In total, 2340 eligible participants in the two areas were randomised to be invited to attend either a commercial pathology centre or a dedicated clinic (see Randomisation section below).

Eligible 45 and Up Study cohort members were sent a postal invitation to take part in the Link-Up Study, which included a covering letter from The 45 and Up Study, a participant information leaflet and a brief questionnaire. Participants randomised to the commercial pathology arm also received a Pathology Request Form, which provided collection instructions for the phlebotomist (quantity, preservative etc.); the participant was asked to bring this form with them when they attended the pathology collection centre.

#### Self-administered questionnaire and consent

Participants were instructed to complete the questionnaire prior to attending their designated collection site and return it when they attended to give a blood sample. The questionnaire was one A4 page, and repeated selected questions from The 45 and Up Study baseline questionnaire. Questions included the participant’s date of birth, self reported height and weight, self-rated health, health events in the last 3 years, and the Medical Outcomes Score Physical Functioning scale.

The consent form was on the reverse of the questionnaire. Participants gave consent for the collection, long-term storage and use of their blood sample for unspecified health research, including genetic research. Participants gave their consent on the understanding that they would not receive any results from tests on their blood sample. Additionally, participants agreed to their questionnaire answers and biomarker data being combined with the health information that was already part of The 45 and Up Study, including linkage to various population databases [3].

#### Dedicated clinics

The dedicated clinics for this trial were set up in a Police, Citizens, Youth Club in Wagga Wagga and a Returned and Services League club in Parramatta, using methods previously employed in a population-based survey study of diabetes and related risk factors [4]. These sites were chosen as they were familiar to the local community and easily accessible. Each location was staffed by a project manager, three casual field staff and a phlebotomist, who were all specifically trained to take the measurements and samples in accordance with the study protocol.

Potential participants randomised to attend a dedicated clinic were given a provisional appointment time in their invitation letter, which they could change if required. Individuals randomised to attend fasting were provided with fasting instructions (*i.e.* no food or drink for 10 hours prior to their appointment, except for water). All fasting appointments were made in the morning to minimise the risk of ill effects and inconvenience to the participant. Clinic staff phoned participants 1–2 days prior to the scheduled appointment to confirm the participant’s attendance. Where a participant was unable to make the scheduled appointment, clinic staff followed-up the participant by phone to reschedule the appointment. Participants were able to attend the dedicated clinics from Sunday to Thursday.

#### Commercial pathology centres

The commercial pathology arm utilised the existing infrastructure of a commercial pathology service with a network of collection centres covering much of New South Wales. Collection of blood samples was undertaken by existing pathology centre staff who were specifically trained by Link-Up Study staff to measure height, weight, waist circumference, blood pressure and heart rate. While each pathology collection centre has its own anthropometric measurement equipment, for consistency the same measurement equipment used in the dedicated clinic arm was supplied for use in the pathology arm. Participants were otherwise able to make use of the existing facilities at these centres (e.g. waiting rooms, phlebotomy chairs, vaccutainers).

The pathology centres are attended by general patients as well as trial participants. Due to the existing workload from fasting general patients, the commercial pathology centres were not able to guarantee that fasting trial participants would be able to be seen in the morning. For this reason, fasting samples were not collected in the pathology centre arm of the trial.

Appointments were not given to participants in the pathology arm. Instead, participants were able to attend at a time convenient to them within a specified 4 week date range. Participants were able to attend the commercial pathology from Monday to Thursday. Potential participants randomised to the reminder arm were sent a second invitation 2 weeks after the initial invitation, if they had not attended by that date.

#### Measurement of height, weight, waist circumference, blood pressure and heart rate

Height was measured using a Charder HM200P stadiometer and was recorded to the nearest 0.1 cm. Participants were instructed to remove their shoes, stand up straight with weight evenly distributed on both feet and position their head so that the line of vision was at right angles to the body. The measurement was taken as the participant inhaled deeply and stretched to their fullest height.

Weight was measured using a Charder MS-3200 digital scale and was recorded to the nearest 0.1 kg. Participants were measured in light clothing and without shoes. Participants were instructed to stand over the centre of the scales with their weight distributed evenly on both feet.

Waist circumference was measured using a Seca 203 waist circumference measuring tape. Participants were instructed to remove any items such as belts or bulky clothing that would interfere with the measurement. Measurements were taken with the participant standing with their feet separated between 25–30 cm and weight distributed evenly on both feet. The tape was positioned on the participant’s waist and the measurement was taken at expiration.

Blood pressure and heart rate were measured using an Omron Digital Automatic Blood Pressure Monitor HEM-907. Participants were instructed to remove any bulky items of clothing around their arms. Prior to the measurements being taken, the participant sat still for 5 minutes with their legs uncrossed and feet flat on the ground.

#### Blood specimen collection and processing for storage

The phlebotomists at each site took a 30 ml blood sample from participants. The sample was collected into tubes as follows:

(i)3 x 6 ml Ethylenediaminetetraacetic (EDTA);

(ii)1 x 6 ml Acid-citrate-dextrose (ACD); and

(iii)1 x 6 ml Lithium Heparin (LiHep).

Immediately following collection each tube was labelled with the date and time of collection, the participant’s date of birth, sex and their unique trial ID number. Samples were refrigerated at 4°C prior to and during transportation to the central laboratory at Darlinghurst, New South Wales. Samples were transported to the laboratory within 36 hours of collection and were processed within 48 hours.

Samples were centrifuged at 2500 rpm for 10 minutes at room temperature. Plasma was removed using a transfer pipette and 0.5 ml aliquotted into 1.0 ml cryo tubes. Buffy coat was then removed into 1.0 ml cryo tubes. One 0.5 ml aliquot of red cells was removed from one of the EDTA tubes, with a transfer pipette into one 1.0 ml cryo tube. Processing generally yielded a total of 22 aliquots for each 30 ml sample (16 plasma, 5 buffy coat, 1 red cells). Once processed, the samples were frozen at −80°C in ultra low temperature freezers.

### Randomisation

**Figure 1:**
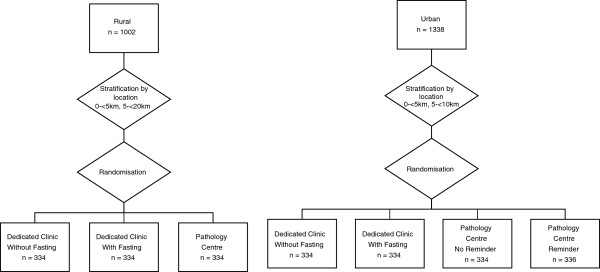
Protocol for allocation of study participants to groups.

A total of 2340 individuals who had provided an address in either Wagga Wagga or Parramatta were randomly sampled from The 45 and Up Study cohort and invited by post to participate in the Link-Up Study (Figure 1). Mailing of invitations began on 25 June 2009, with data collection from 5 July 2009 to 6 August 2009 in Wagga Wagga and from 16 August 2009 to 24 September 2009 in Parramatta.

The addresses of 45 and Up Study participants have been geocoded using the geocoding system in Freely Extensible Biomedical Record Linkage (FEBRL) [5]. The General Post Office (GPO) was selected as the central area at each location and distance from the central area was calculated using the great-circle distance formula. Participants with a residential address geocoded to the street level, and within 10 km of Parramatta GPO (33.81S, 151.00E) or 20 km of Wagga GPO (35.10S, 147.37E) were selected into the sampling frame. A stratified sample of potential participants was drawn randomly (using a computer generated randomisation list) in each area, with stratification by distance from the GPO.

In Wagga Wagga, 1002 participants were selected, stratified by distance (0-5 km from GPO, 5-20 km from GPO) with equal allocation into each stratification group. In Parramatta 1002 participants were initially selected, stratified by distance from Parramatta GPO (0-5 km from GPO, 5-10 km from GPO).

From the samples in each area, participants were randomly allocated in equal proportions to one of three groups (using a computer generated randomisation list):

(i)invited to attend the dedicated clinic, not fasting;

(ii)invited to attend the dedicated clinic, fasting; and

(iii)invited to attend the pathology centre, not fasting, no reminder letter.

After preliminary results of relatively low response rates were available from Wagga Wagga, a decision was made to include a fourth group in the trial in Parramatta: invited to attend the pathology centre, not fasting, with reminder letter. An additional 336 eligible participants in the Parramatta area that had not already been selected for sampling were randomly selected (using a computer generated randomisation list) and allocated to this additional pathology arm.

KDR was responsible for generating the syntax used to randomly sample and allocate participants to treatment groups. Randomisation and stratification processes are summarised in Figure 1.

### Blinding

Although invitees were told in the study information leaflet that they were part of a pilot project and that their participation would assist “in deciding the best way of implementing the Link-Up Project”, they were not aware of the trial hypotheses or randomisation to differing data collection site types, fasting status and reminder letter receipt. In analysing and interpreting the data, the authors were aware of the randomisation status of study participants.

### Outcome measures

The primary outcome measure for this trial is participation in the Link-Up Study, through attendance at a biospecimen collection site to provide a blood sample and giving written consent to participate, including use of blood samples for genetic and other research. The proportion of invitees who went on to participate in this way in the Link-Up Study is termed the “response rate”.

Concern is often expressed that dedicated clinics and highly trained study staff are necessary to make physical measurements for cohort studies and that staff from health services may not be capable of taking measurements in the same way. Among individuals who went on to participate in the Link-Up Study, the biospecimen yield and features of the biomarker data (height, weight, heart rate and systolic and diastolic blood pressure) were compared for those attending the dedicated clinic and those attending the pathology centre, in order to investigate whether there was any empirical evidence of systematic differences that might indicate issues with data quality.

### Statistical methods

We estimated we would need 668 participants in each treatment group across the area, based on an initially estimated response rate of 50% and difference in response rate of 7.6% between treatment groups for power of 0.796. We allocated 334 persons to each area and planned to pool across areas to compare treatment effects of data collection type (pathology vs. dedicated clinic).

Data from the participants’ 45 and Up Study baseline questionnaire were added to the data on responses and clinical data from the Link-Up Study. Information from the Link-Up Study included: area (Wagga Wagga or Parramatta); distance from GPO (categorised as <5 km and >5 km); and the trial group that the participant was allocated to. Distance from GPO was based on the most current residential address held by The 45 and Up Study.

Response rate was analysed using a log binomial model (proc genmod in SAS, v9.2 Cary NC, USA) with response (yes or no) as the outcome, and randomisation group as the predictor variable. Associations with exposure variables were expressed as a participation rate ratio (RR). Quantifying differences in response rate between randomisation groups, stratified by area. Linear combinations of coefficients were used to test three specific hypotheses about differences in response rates between:

(i)fasting versus non-fasting groups in dedicated clinics;

(ii)reminder versus no reminder, for specimens collected at the pathology service at Parramatta only;

(iii)dedicated clinic versus pathology service.

Wald 95% confidence intervals were calculated for rate ratio estimates.

Among those participating in the Link-Up Study, the means, standard deviations and differences between self-reported and measured height and weight and measured blood pressure were compared between dedicated clinic and pathology service, within each area, using standard t-tests and Bland-Altman plots. Biospecimen yields were measured according to the number of aliquots of plasma, buffy coat and red cells in each preservative type, and compared with pre-specified targets for each of these biospecimens and also compared between the different collection sites, using standard t-tests.

We have not made a formal correction (e.g. Bonferonni correction) for multiple testing (multiple treatment comparison and multiple outcomes) in our study. We have calculated five p-values for the main outcome (response rate), 14 p-values for measurement differences, and 16 p-values for blood aliquot yield. The chance of a Type I error increases with the number of tests performed, so our results should be interpreted with the number of tests for each outcome in mind.

Ethical approval for the study was provided by the University of New South Wales, Human Research Ethics Committee, the Australian National University Human Research Ethics Committee, and the University of Western Sydney Human Research Ethics Committee.

## Results

### Baseline characteristics according to randomisation group

Table 1 shows a range of demographic, lifestyle and other characteristics of the trial participants according to the group they were randomised to. There were no marked differences between the groups, in either urban or regional strata.

**Table 1:** Participant characteristic by area and randomisation status

**Wagga Wagga Randomised to:**	**Group 1 Dedicated clinic not fasting**	**Group 2 Dedicated clinic fasting**	**Group 3 Pathology centre not fasting no reminder**	
Total (N)	334	334	334	
Age (mean [SD])	60.8 [9.7]	61.6 [10.5]	60.7 [10.3]	
Sex (% male)	47.6	45.5	40.7	
Distance from centre (mean km [SD])	5.0 [2.9]	5.0 [3.3]	5.2 [3.3]	
Work status (% full time paid work)	31.1	29.6	33.2	
Time since recruitment (% >1 year)	48.5	52.1	52.1	
Education (% tertiary educated)	22.9	24.7	19.7	
Carer status (% full time carer)	3.6	3.0	4.5	
Disability (% major disability)	3.8	7.3	4.9	
Physical function (% severe limitation)	11.8	14.5	14.7	
Smoking (% current smokers)	7.5	5.5	6.6	
BMI (% obese (BMI ≥ 30 kgm^-2^))	27.1	23.5	30.2	
**Parramatta Randomised to:**	**Group 1 Dedicated clinic not fasting**	**Group 2 Dedicated clinic fasting**	**Group 3 Pathology centre not fasting no reminder**	**Group 4 Pathology centre not fasting with reminder**
Total (N)	334	334	334	336
Age (mean [SD])	64.2 [12.0]	63.1 [12.7]	64.1 [11.8]	63.5 [11.2]
Sex (% male)	47.6	49.7	50.6	49.4
Distance from centre (mean km [SD])	5.7 [2.4]	5.7 [2.6]	5.7 [2.5]	5.8 [2.5]
Work status (% full time paid work)	28.7	31.8	28.2	29.6
Time since recruitment (% >1 year)	53.1	47.7	54.2	55.1
Education (% tertiary educated)	28.3	25.7	25.6	23.1
Carer status (% full time carer)	3.3	4.5	5.1	4.2
Disability (% major disability)	10.5	5.9	4.0	6.6
Physical function (% severe limitation)	18.6	17.8	18.4	21.1
Smoking (% current smokers)	7.3	8.4	6.9	5.7
BMI (% obese (BMI ≥ 30 kgm^-2^))	24.9	22.5	21.8	22.3

### Overall response to the invitation to participate

The overall uptake of the invitation to participate in the Link-Up Study was 33% (762/2340). The response rate was 41% (410/1002) among participants resident in the regional area (Wagga Wagga) and 26% (352/1338) among those resident in the urban area (Parramatta)(p < 0.0001).

### Response rate according to whether participants were invited to attend fasting or not fasting

Individuals invited to provide biospecimens and physical measures at the dedicated clinics were randomly allocated to be invited fasting or not fasting. In Wagga Wagga, a total of 46% of those randomised to attend the clinic in a non-fasting state attended their appointment, compared to 47% randomised to fasting (participation rate ratio, RR fasting versus non-fasting  = 1.01, 95% CI 0.91-1.11, p = 0.87; Table 2). Figures for non-fasting and fasting at Parramatta were 31% and 30%, respectively. (RR = 0.99, 95%CI 0.86-1.13), p = 0.88; Table 2). Combining the dedicated clinic in both areas, the response rate was 38% (257/668) not fasting and 38% fasting (257/668) (RR = 1.00, 95%CI 0.91-1.08, p = 0.98).

**Table 2:** Response rate to participation in the Link-Up Study among existing participants in The 45 and Up Study, by area of residence and randomisation status

**Group**	**Total**	** Participation rate ratio (RR)**		**P (*χ***^**2**^**test)**
	**N**	**n (%)**	**RR (95% CI)**	
**Total**	2340	762 (33%)		
**Wagga Wagga**	1002			
Group 1 Dedicated Clinic without fasting	334	154 (46%)	1.00	
Group 2 Dedicated Clinic with fasting	334	156 (47%)	1.01 (0.91-1.11)	Fasting vs. non fasting: p = 0.87
Group 3 Pathology Service no reminder	334	100 (30%)	0.64 (0.53-0.79)	Clinic vs. pathology service: p <0.01
**Parramatta**	1338			
Group 1 Dedicated Clinic without fasting	334	103 (31%)	1.00	
Group 2 Dedicated Clinic with fasting	334	101 (30%)	0.99 (0.86-1.13)	Fasting vs non fasting: p = 0.88
Group 3 Pathology Service no reminder	334	72 (22%)	0.69 (0.53-0.90)	Clinic vs pathology service: p <0.01
Group 4 Pathology Service with reminder	336	76 (23%)	1.01 (0.93-1.09)	Reminder vs no reminder: p = 0.74

### Response rate according to whether or not participants received a reminder letter

Participants invited to attend the Wagga Wagga pathology centre did not receive a reminder letter. Individuals invited to attend the Parramatta pathology centre were randomised to either receive or not receive a reminder letter, if they had not responded to their initial invitation in the first two weeks of data collection. The response rate was 22% among individuals randomised to attend the Parramatta pathology service without a reminder and 23% among those who were sent a reminder letter (RR reminder versus no reminder  = 1.01, 95% CI 0.93-1.09, p = 0.74).

### Response rate according to whether participants were invited to attend a dedicated clinic or commercial pathology centre

In Wagga Wagga, the response rate was 46% among those randomised to attend the dedicated clinic, not fasting and 30% among those randomised to attend the pathology centre, not fasting (RR = 0.64, 95% CI 1.0.53-0.79, p < 0.01; Table 2). In Parramatta, the response rate for those randomised to attend the dedicated clinic not fasting was 31% compared to 22% among those randomised to attend the pathology centre not fasting, with no reminder (RR = 0.69, 95% CI 0.53-0.90, p < 0.01). Combining the regional and urban areas, the fasting and non-fasting participants and those who did and did not receive a reminder letter, the overall response rate was 38% (514/1336) at the dedicated clinics and 25% (248/1004) at the pathology centres (RR = 0.67, 95% CI 0.56-0.78, p < 0.01).

For the randomised elements of this study relating to fasting status and the reminder letter, a meaningful difference in the RR can effectively be ruled out, since they are estimated with high precision. Hence, there is little evidence for large differences in these RR.

### Physical measures and biospecimen yields at dedicated clinics and commercial pathology centres

Mean height, weight and systolic blood pressure did not differ significantly between the dedicated clinics and commercial pathology centres in either the regional or urban areas (Table 3). Diastolic blood pressure was significantly higher at the Wagga Wagga pathology centre than at the dedicated clinic (p < 0.01), but did not differ significantly between the two types of collection sites in Parramatta. The differences between measured height and weight, and self-reported height and weight on the brief questionnaire completed prior to measurement, were similar across all four sites. Bland-Altman plots suggested no large or systematic variation in the differences between self-reported and measured height and weight according to the type of data collection site (data not shown).

**Table 3:** Comparison of height, weight, blood pressure and heart rate measurements at the dedicated clinics versus pathology services

**Area: Wagga Wagga**			
**Measurement**	**Dedicated clinic**	**Pathology service**	**p(Difference)**
Mean			
Height [cm (95% CI)]	168.3 (166.8-169.7)	166.0 (164.3-167.8)	0.710
Weight [kg (95% CI)]	80.2 (78.3-82.1)	76.8 (76.8-84.5)	0.807
Difference between			
self-reported and measured:			
Height [cm (95% CI)]	0.5 (0.2-0.9)	1.2 (0.5-2.1)	0.665
Weight [kg (95% CI)]	−1.6 (−1.8- -1.3)	−1.2 (−1.8- -0.6)	0.842
Mean			
Systolic blood pressure [mmHg (95%CI)]	134.5 (132.6-136.4)	135.6 (131.3-140.1)	0.754
Diastolic blood pressure [mmHg (95%CI)]	75.5 (74.3-76.6)	80.1 (77.9-82.4)	<0.01
Pulse rate [beats per minute (95% CI)]	69.5 (68.2-70.8)	71.8 (69.9-73.6)	0.078
**Area: Parramatta**			
**Measurement**	**Dedicated clinic**	**Pathology service**	**p(Difference)**
Mean			
Height [cm (95% CI)]	166.9 (165.6-168.3)	166.7 (165.1-168.2)	0.808
Weight [kg (95% CI)]	78.6 (76.2-81.0)	77.5 (74.6-80.5)	0.567
Difference between			
self-reported and measured:			
Height [cm (95% CI)]	1.5 (1.1-1.9)	0.5 (0.1-1.1)	0.272
Weight [kg (95% CI)]	−0.9 (−1.2- -0.5)	−1.1 (−1.5- -0.7)	0.339
Mean			
Systolic blood pressure [mmHg (95%CI)]	131.4 (128.9-134.0)	132.2 (129.3-135.0)	0.708
Diastolic blood pressure [mmHg (95%CI)]	76.6 (75.1-78.1)	75.5 (74.3-76.6)	0.312
Pulse rate [beats per minute (95% CI)]	71.5 (70.0-73.1)	69.5 (68.2-70.8)	0.619

Median biospecimen yields and 5^th^ centiles were within one aliquot of the target for each type of sample fraction, preservative and biospecimen collection site, except for the 5^th^ centile at the Wagga Wagga dedicated clinic for EDTA- and Lithium-Heparin-plasma (Table 4). No systematic variation in yield between the dedicated clinics and pathology centres could be identified.

**Table 4:** Comparison of number and types of aliquots collected at the dedicated clinics versus pathology services

**Area: Wagga Wagga**				
**Type of aliquot**	**Target**	**Dedicated clinic**	**Pathology centre**	**p(Difference)**
	number of aliquots	median number of aliquots (5^th^-95^th^ centile)	median number of aliquots (5^th^-95^th^ centile)	median number of aliquots dedicated clinic vs pathology centre
**EDTA**				
Buffy coat	3	3 (2–3)	3 (3–3)	0.40
Plasma	10	12 (8–16)	12 (10–15)	<0.01
Red cells	1	1 (1–1)	1 (1–1)	0.87
**ACD**				
Buffy coat	1	1 (1 – 1)	1 (1–1)	0.74
Plasma	3	5 (2 – 6)	5 (4–6)	<0.01
**Li-Hep**				
Buffy coat	1	1 (1–1)	1 (1–1)	0.07
Plasma	3	4 (1–5)	4 (3–5)	0.01
**Total**	22	26 (18–33)	27 (24–31)	<0.01
**Area: Parramatta**				
**Type of aliquot**	**Target**	**Dedicated clinic**	**Pathology centre**	**p(Difference)**
	number of aliquots	median number of aliquots (5^th^-95^th^ centile)	median number of aliquots (5^th^-95^th^ centile)	median number of aliquots dedicated clinic vs pathology centre
**EDTA**				
Buffy coat	3	3 (3–3)	3 (3–3)	0.38
Plasma	10	12 (9–13)	11 (9–13)	<0.01
Red cells	1	1 (1–1)	1 (1–1)	*
**ACD**				
Buffy coat	1	1 (1–1)	1 (1–1)	*
Plasma	3	4 (3–5)	4 (3–5)	0.06
**Li-Hep**				
Buffy coat	1	1 (1–1)	1 (1–1)	0.16
Plasma	3	4 (3–4)	3 (2–4)	0.01
**Total**	22	26 (21–29)	25 (20–28)	<0.01

* Both groups have the same number of aliquots of this type.

### Adverse events

One episode of fainting occurred in a fasting participant at the Wagga Wagga dedicated clinic. This participant had done a number of hours of hard physical work before attending the collection site. No other adverse events were observed.

## Discussion

The collection of biospecimens and biomakers is increasingly recognised as an important component of large scale epidemiological research. Such data enhance substantially the range and nature of the research questions that can be addressed and allow joint consideration of environmental and genetic factors. However, the establishment of large scale biobanks requires consideration of pragmatic and cost factors, alongside traditional scientific and methodological issues [6].

We found that a single mailed invitation to existing cohort study members to donate a blood sample, have physical measurements taken and have these included in a long term biobank for unspecified research purposes yielded a substantial number of participants, with 33% of those approached taking part. The randomised data indicate that response rates were one-third lower at existing commercial pathology centres than at collection sites established specifically for the purpose of the study, where potential participants were given specified appointment times and phone reminders to attend their appointment. There was no significant difference in response rates according to whether or not participants were randomised to be invited to attend in a fasting or non-fasting state or according to whether or not they received a reminder letter. Those residing in regional areas were more likely to participate than those in urban areas. No systematic differences in data quality and biospecimen yield were seen between the dedicated clinics and pathology centres.

We are not aware of any other studies that have made randomised comparisons of biobank participation rates according to type of collection site, so the evidence to date in this area is limited. There are several plausible explanations for the higher attendance rates at dedicated clinics. Studies researching the effect of invitation type on attendance for breast and cervical cancer screening have established that assigned appointments increased uptake of screening [7-9] and this was the method of choice for recruitment into the UK Biobank [1]. A phone call reminder to follow up a written invitation is an additional effective method of increasing participation [10,11]. Furthermore, several studies have found that establishing a separate clinic in general practice for cancer screening and vaccinations is an effective way of increasing response rates [8,12,13], however whether this finding is generalisable to recruitment to biobanks is unclear. The dedicated clinics were able to accommodate weekend recruitment, which was not possible through the pathology centres.

There is little research that can explain why people may be willing to participate in a biobank in some situations, but not others. Previous studies indicate that multiple methods of contact with participants leads to higher response rates in studies in general [10,11]. Other studies have found that multiple reminders are an effective way of increasing response rates to postal questionnaires [14,15]. However, in the current trial, a single reminder letter sent to half the cohort members randomised to attend a pathology centre in Parramatta did not have a significant effect on response rates. The reason for this is not known; potential explanations include the fact that only one reminder was given, that the reminder was also sent by mail, that study power was limited or that no genuine difference was present, in this setting.

Research has previously been undertaken to investigate whether individuals would be willing to participate in biobanking, using hypothetical scenarios [16-19], however, data comparing the factors related to actual participation, are limited. The results from the present trial indicated that response rates were higher amongst participants in the regional area than in the urban area. A large multicentre US study found that enrolment site was the most important influence on consenting to donate biospecimens, with response rates varying between 40% and 100% over the 24 enrolment sites used in the study [20]. However, although it was noted that the sites represented a diverse range of urban and regional areas, the researchers did not indicate which locations yielded higher response rates. In contrast, another biobank study found no significant difference based on residential area, however this study measured willingness to participate and not actual participation [21].

There are a number of potential limitations of the current trial that should be borne in mind. We used address details from the baseline mailout for The 45 and Up Study cohort, updated where participants notified The 45 and Up Study Coordinating Centre of a change of address. However, it is likely that some participants had moved, without updating their address details, in the one to three years since joining The 45 and Up Study; others may not have received their invitation or may have been unavailable to take part in the Link-Up Study for some other reason (e.g. holidays, illness). Although the randomised design means that this is unlikely to bias the main comparisons, it does mean that the response rate should be considered to be the proportion of people taking part in the biobank as a proportion of those sent invitations, not as a proportion of those who actually received an invitation. The trial only compared two models of biospecimen and physical measurement data collection. It is therefore not possible to separate different elements of the two designs. For example, it is not possible to establish what the separate contributions of the specific appointment time, weekend appointments and the phone reminders were to response rates, since these were all part of the general dedicated clinic protocol.

The trial examines response rates to biospecimen collection and physical measurements among people already participating in The 45 and Up Study. Similar to most cohort studies, The 45 and Up Study is not designed to be representative of the general population [3]. Rather, it aims to provide a large cohort of individuals consenting to comprehensive and long term follow up, with sufficient numbers in a range of population and exposure groups to allow for valid internal comparisons. The response rate to the main cohort was ~18%. The overall response rate to the Link-Up Study of 33% could be interpreted broadly as 6% of a general population study taking part; comparable to the 9% seen in the UK biobank, but less than other more localised community-based cohort studies (e.g. EPIC Norfolk, Framingham).

The Link-Up Study aimed to test potential strategies for large scale collection of biospecimens and physical measurements from The 45 and Up Study participants. While there was some limited promotion of the trial in the annual newsletter distributed to 45 and Up Study participants (via post or e-mail) and on the website of The 45 and Up Study, there was no media promotion of the trial or local publicity, or other strategies which could potentially improve local participation. This is because the trial sought to explore methods that could be used for The 45 and Up Study as a whole and there would be potential difficulties doing this across the ~800,000 km^2^ of NSW, for a prolonged period of time. It shows the response rate to a single ‘cold’ invitation to take part, with reminders. There remains considerable potential for improving response rates.

Dedicated clinics are considered the gold standard for collection of biospecimens and biomarker data for cohort studies, but are relatively expensive and time intensive to establish. Funding must be sourced for infrastructure such as premises and equipment and qualified staff must be hired and trained; these costs will be greater when sampling a geographically dispersed population. In addition to cost, there are logistical implications associated with establishing a clinic *de novo*. Once established, they can generally only accept participants for a restricted time period.

Collection through existing pathology centres has the advantage of being easier to implement, and having a broad network of collection locations that can be utilised fairly rapidly. Disadvantages are that pathology centres are less flexible and study design may need to be adapted to this; for example, large numbers of fasting participants, designated appointment times and weekend collection may not be able to be accommodated. While response rates were 50% higher at dedicated clinics, compared to commercial pathology centres, the total weighted costs in this trial were 110% higher per invitee, and 40% higher per final participant, at the dedicated clinic versus the pathology centres.

The 45 and Up Study cohort is spread across NSW, with over 50% in rural and regional areas. It would not be practical to set up dedicated clinics to serve the entire cohort, especially in sparsely populated areas. Hence, in this case, a mixed method of collection is likely to be the most practical.

It is generally assumed that more specialised staff, experienced in the specific type of data collection entailed, will collect data that is of higher quality than less specialised or experienced staff. However, the assumption has not, to our knowledge, been tested empirically in the setting of a biobank. The dedicated clinics were set up for the sole purpose of collecting blood samples for research, in accordance with the trial protocol. Conversely, the pathology centres provide a general commercial service, collecting a range of biospecimens for various purposes (predominantly clinical) and according to different instructions. The pathology staff relied on trial participants bringing the Pathology Request Form with them, which provided specific instructions to the phlebotomist. We found no material differences in the physical measurements taken between the dedicated clinics, with more specialised staff, and those at the pathology centres, and biospecimen yields were close to or exceeded targets for the vast majority of participants. This suggests that specialisation beyond the basic training received by pathology centre staff for the purposes of this trial did not appear to be accompanied by a discernable improvement in data quality. If rolled out on a larger scale, specific training of staff at every commercial pathology centre may be impractical and the incidence of protocol deviations could potentially be greater, as there would be less awareness of the study amongst staff in a broad network of pathology centres. Hence, the findings observed here may represent a best-case scenario.

## Conclusions

For studies requiring the collection of biospecimens, use of dedicated clinics set up specifically for the purpose of the study, with designated appointment times, can increase response rates when compared to using existing pathology centres. However, each collection method has advantages and disadvantages, including the fact that the cost of collection through these dedicated clinics is greater. Data quality and average biospecimen yield were similar for the different types of data collection sites, within this trial.

## Abbreviations

ACD: Acid-citrate-dextrose; CI: Confidence interval; EDTA: Ethylenediaminetetraacetic; FEBRL: Freely extensible biomedical research linkage; GPO: General Post Office; LiHep: Lithium Heparin; MOS-PF: Medical Outcomes Score-Physical Functioning scale; RR: Rate ratio.

## Competing interests

The authors declare that they have no competing interests.

## Authors’ contributions

Study conception and design: EB, LJ. Study coordination and data collection: NH, EB. Biostatistical supervision and conduct of analyses: KR. Interpretation of analyses: EB, LJ, KR. Literature review: TM, NH. Drafting of manuscript: EB, NH, TM. Revision of manuscript for important intellectual content: NH, KR, TM, LJ. All authors read and approved the final manuscript.

## Pre-publication history

The pre-publication history for this paper can be accessed here:

http://www.biomedcentral.com/1471-2288/12/147/prepub
